# The Research Progress of Host Genes and Tuberculosis Susceptibility

**DOI:** 10.1155/2019/9273056

**Published:** 2019-08-14

**Authors:** Li Cai, Zhan Li, Xuhua Guan, Kun Cai, Lei Wang, Jiafa Liu, Yeqing Tong

**Affiliations:** ^1^Wuhan Center for Disease Control and Prevention, Wuhan 430015, China; ^2^School of Health Sciences, Wuhan University, Wuhan 430071, China; ^3^School of Public Health, Tongji Medical College, Huazhong University of Science and Technology, Wuhan, China; ^4^Hubei Center for Disease Control and Prevention, 430079, China

## Abstract

**Background/Aims:**

Nucleotide diversity may affect the immune regulation of tuberculosis (TB) patients, leading to the individual susceptibility to TB. In recent years, there are a lot of researches on the association of host genetic factors and TB susceptibility which has attracted increasing attention, and the in-depth study of its mechanism is gradually clear.

**Materials:**

We made a minireview on the association of many candidate genes with TB based on recent research studies systematically, such as the human leukocyte antigen (HLA) gene, the solute carrier family 11 member 1 (SLC11A1) gene system, the vitamin D receptor (VDR) gene, the mannan-binding lectin (MBL) gene, the nitric oxide synthase 2A (NOS2A) gene, the speckled 110 (SP110) gene, and the P2X7 receptor (P2X7) gene. The discovery of these candidate genes could reveal the pathogenesis of TB comprehensively and is crucial to provide scientific evidence for formulating the related measures of prevention and cure.

**Discussion:**

The host genes play important roles in the development of TB, and the host genes may become new targets for the prevention and treatment of TB. Effective regulation of host genes may help prevent or even treat TB.

**Conclusion:**

This minireview focuses on the association of host genes with the development of TB, which may supply some clues for future therapies and novel drug targets for TB.

## 1. Introduction

Tuberculosis (TB), an important chronic infectious disease caused by mycobacterium tuberculosis (MTB), which is seriously threatening to human health, is one of the top ten causes of death in the world. About one-third of the world's population is infected with MTB according to the statistics [1, 2], in which only about 1/10 fall ill [3], and few of them have clearly identifiable risk factors, such as diabetes, advanced age, alcoholism, HIV infection, or the use of corticosteroids; this suggests that a significant proportion of these individuals may be naturally resistant to TB infection; in addition to environmental, pathogen, and socioeconomic factors, host genetic diversity plays a nonnegligible role in the initiation of TB. Candidate gene studies and genome-wide association studies (GWAS) have revealed many genetic polymorphisms related to the occurrence and prognosis of TB.

Researches on genetic variants that confer susceptibility to TB had indicated that the genetic components determining susceptibility are assigned by several small predisposing genes rather than a single major type. To date, abundant research studies have identified and explored the specific genes susceptible to TB, which are divided into two categories: HLA genes and non-HLA genes.

## 2. HLA Genes

Human leukocyte antigens (HLAs) are the most complex genetic polymorphism system known to humans. The HLA gene is located on the short arm of the 6th chromosome, is about 4000 kilobases (kb) in length, contains 224 loci, and encodes a series of complicated markers on the surface of the cell membrane. And the HLA gene complex is distributed into three regions: the HLA-I gene is located in the telomere end of the chromosome and is divided into -A, -B, and -C genes. HLA-I molecules combining with endogenous antigens are displayed on the cell surface and are recognized by CD8^+^T cells. The HLA-II gene is close to the centromere, mainly including DQ, DR, and DP subregions. HLA-II molecules bind and then present exogenous antigens to the CD4^+^ T cell. The HLA-III gene is located between the HLA-I gene and the HLA-II gene. HLA-III molecules do not participate in the antigen presentation except that a minority of them can participate in the immune regulation through the complement pathway. In general, it can be concluded that HLA-I and HLA-II genes are mainly responsible for antigen presentation and regulation of immune responses, which plays an important role in individual susceptibility to disease [4].

A great number of studies have explored the relationship between HLA genes and TB susceptibility, which is summarized in Table 1. Researches on the association between polymorphisms of HLA genes and TB in different populations have found that because of HLA loci polymorphisms, the linkage disequilibrium of genetic loci and their complex relationships with cytokines, it was impossible to draw unanimous conclusions among different ethnic groups, and it was also an arduous task to explain the association of HLA loci with diseases. And recent studies have focused on non-HLA genes [5].

## 3. Non-HLA Genes

### 3.1. VDR Gene

Epidemiological studies have shown that vitamin D deficiency is associated with susceptibility to TB [22, 23]. Vitamin D works through the vitamin D receptor (VDR). The VDR gene has an important effect on both congenital and acquired immunity; bacteria entering the lungs are engulfed by macrophages; VDR and its activated form, 1,25-dihydroxyvitamin D3, directly stimulate induction of cathelicidin (LL-37) though the vitamin D-dependent pathway, killing intracellular MTB [24, 25]; and 1,25-dihydroxyvitamin D3 can also induce human monocyte autophagy through antimicrobial peptides [26], then exert various immune regulation effects, but its role in acquired immunity is still controversial.

A large number of studies have shown that VDR polymorphisms are associated with susceptibility to TB, but only in Asian populations [27, 28]. The results of association studies between VDR gene polymorphisms and susceptibility of TB are inconsistent with different regional differences. In the Gujarati population of India, the TaqI TT genotype and the FokI ff genotype are risk genotypes [29]; nevertheless, VDR-(FF+ff) and tt genotypes are TB risk genes and VDR-(TT+Tt) and Ff genotypes are antagonistic genes that protect the body against TB in a Ningxia population of China [28]. A recent meta-analysis of twenty-three studies found a correlation between VDR BsmI and Fok1 genotypes and TB in the Asian population, but there was no association in African and South American populations [30].

### 3.2. SLC11A1 Gene

The solute carrier family 11 member 1 (SLC11A1)/natural resistance-associated macrophage protein 1 (NRAMP1), initially found in mice, is a crucial determinant susceptible gene of TB in mice and is also the most widely studied candidate gene for TB susceptibility in non-HLA genes. After macrophage activation, NRAMP1 can transport divalent captions through the phagocytic membrane, thereby affecting the growth of MTB in phagosome.

There are series of findings indicating that the polymorphisms of the SLC11A1 gene are associated with the susceptibility of TB in different populations. Studies have confirmed that 118 allele (GT) 9 in the promoter region can lead to the high expression of the SLC11A1 gene and to building immunity against TB, whereas 120 allele (GT) 10 has weaker promoter activity and less obvious susceptibility to TB [31]. Inversely, research has also been carried out on the 3′ untranslated region (3′UTR) of the SLC11A1 gene in West African, Asian, and South African populations [32], indicating that the SLC11A1 3′UTR mutation can significantly increase the risk of pulmonary TB. Moreover, a study of the SLC11A1 gene in African-American and American Caucasian populations indicates that there are two TB susceptibility SNPs—rs17221959 and rs3731863—in the Caucasian population, while rs3731865 is susceptible to TB in the African-American population; nevertheless, these SNP loci do not appear in the (GT)9 repeat sequence allele, the TGTG deletion, and the 3′UTR of the SLC11A1 gene promoter region [33]. Several other studies have also pointed out that the SLC11A1 gene not only is associated with TB susceptibility and progression of pulmonary TB but may also play a role in the emergence and development of drug-resistant TB [34].

### 3.3. MBL Gene

Mannose-binding lectin (MBL) belongs to the Ca^2+^-dependent lectin family and is composed of polymers consisting of the same 32 kDa polypeptide chains. There are two kinds of human MBL genes—the MBL1 gene and the MBL2 gene; the latter is a functional gene which encodes MBL proteins. The difference in serum MBL levels among individuals is mainly caused by the point mutations in the MBL2 gene exon code 52 (rs5030737), 54 (rs1800451), and 57 (rs1800450). The MBL gene is essential for the body to resist pathogen invasion.

Population-related association studies in South Africa, Denmark, and Turkey have also shown that the three polymorphisms of mutations talked above are associated with MTB infection, especially in patients with brain TB [6]. Analogously, polymorphism of the MBL gene in the Chinese Han population may also be significantly correlated with pulmonary TB susceptibility [35]. The interaction of NRAMP1, VDR, and MBL is related to the susceptibility to TB, but the relationship between genes remains unknown [36].

### 3.4. NOS2A Gene

The nitric oxide synthase 2A (NOS2A) gene regulates the NO level by encoding three synthases (neuronal, inducible, and endothelial NOS) that mediate immune responses to TB and other infectious diseases.

In a study from the Brazilian population, two functional polymorphisms rs2779249 and rs2301369 located in the promoter region of the NOS2A gene are associated with TB susceptibility, but the polymorphic locus rsl800482, which is also located in the same promoter region, does not have any relationship with TB in the Mexican population [37]. In a South African population, the haploid formed by rs9282799 and rs8078340, two functional sites in the NOS2A promoter region, is also found correlated with susceptibility to pulmonary TB [38]. Recently, studies have found that rs2274894 and rs7215373 have significant association with the susceptibility of TB in African-American and non-Caucasian populations [39].

### 3.5. SP110 Gene

In the study of a mouse model, the Iprl gene was found to resist pathogen infection and regulate the natural immunity of TB by controlling the proliferation of tubercle bacilli in the lung and the apoptosis of macrophages. In the human body, the speckled 110 (SPll0) gene is homologous to the Iprl gene; it is supposed that the SP110 nucleus protein may mediate the interaction between the host and pathogen by participating in the transcription activation of macrophages to intracellular pathogens. Therefore, it is speculated that the SPll0 gene is a candidate gene for susceptibility of TB. It has been found that three single nucleotide polymorphisms (SNPs) of SP110 are susceptible gene polymorphisms of TB in West African populations [40]. In a Chinese population, in addition, the polymorphisms of the SP110 genes rsll35791, rs722555, and rsll679983 are also associated with TB susceptibility. However, subsequent experiments in the Ghanaian, Russian, or South African populations fail to yield the same results [41]. Despite that the association of the SP110 gene with TB is also racially specific and needs further confirmation, given its impact on host TB immunity, studies have suggested that SP110b can serve as a potential target for host-directed therapy [42].

### 3.6. TLR Gene

The Toll-like receptor (TLR) family belongs to pattern recognition receptors that are capable of identifying various pathogens, which plays an important role in early immune recognition and inflammatory response, and is of great significance to innate and adaptive immune responses. The relevance between the TLR gene and TB susceptibility is summarized in Table 2. These data indicate that TLR polymorphism is significantly associated with susceptibility to TB and seems to be of high diversity among different ethnic groups.

### 3.7. P2X7 Gene

The P2X7 receptor is a cationic channel presenting on the surface of blood cells and immune system cells and is especially highly expressed in the surface of macrophages. The P2X7 receptor can be activated by extracellular ATP, causing specific cationic channels to open, leading to Ca^2+^ and Na^+^ influx and K^+^ outflow and ultimately to cell apoptosis or tubercle bacillus death.

At present, the most studied 1513A-C locus variant in the P2X7 gene has discrepant verdicts in different races and regions. Research in the Gambian population shows that the 1513 A-C (rs3751143) variation is not associated with TB susceptibility, while the study showed that the 762C locus has a significant protective association against TB [51]. A study on Southeast Asian refugees in Australia had found that 1513 SNP is not related to TB, but 762C-locus diversity is significantly correlated with pulmonary TB [52]. A recent study shows that rs1718119 was related to a reduced risk for all active TB and sputum smear-positive cases in the Chinese Han population, and rs7958311 may contribute to a successful treatment outcome [53].

### 3.8. CD209 Gene

The human CD209 gene encodes DC-SIGN, a kind of adhesion molecule expressed on humandendritic cells (DCs), belongs to the type-II transmembrane C-type lectin that recognizes Lewis-X glycosyl, mannose polymer, and mannose cap end and mediates DC-T cell interactions, which is associated with DC migration. DC-SIGN is also expressed on alveolar macrophages of TB patients and is an important pattern recognition receptor (PRR) of MTB, which means it may be the main pathway for MTB to enter DC. Moreover, DC-SIGN is the target of Man-LAM toxicology. DC-SIGN may play an immunosuppressive effect by interfering with the TLR2/TLR4 pathway. Two promoter variants in sub-Saharan Africa were first reported in 2006—SNP rs4804803 (-336A/G) and rs735239 (-871A/G)—that were associated with TB susceptibility [54]. The newest meta-analysis also confirmed that the -871A/G is associated with TB immune protection in all populations and that the -336A/G is a risk factor for TB only in Asian populations [55]. Whereas other studies support that the -336A/G has a protective effect against TB infection in sub-Saharan Africa [56], CD209-encoded DC-SIGN is a MTB receptor on DCs and previous in vitro studies have shown that -336G alleles can result in downregulated CD209 mRNA expression, which means the -336G variant has lower basal expression of CD209 when compared to the -336A ancestral allele, so a decrease in DC-SIGN receptor levels may have a protective effect on most TB; conversely, MTB may subvert the immune response by the DC-SIGN receptor [54].

### 3.9. Cytokines and Chemokines

Immune response is regulated by the interaction of lymphocytes, antigen presenting cells, and cytokines secreted by these cells. A growing number of studies have proved that gene polymorphisms in the promoter region or cytokine gene coding region are the host factors affecting susceptibility of TB.

Chemokine, a kind of protein with low molecular weight which guides white blood cells to specific sites of infection, plays a crucial role in combating MTB infection. When an organism gets infected, MTB induces macrophages to produce excess CC-chemokines (including MCP-1, CCL2, MIP-1*α*, CCL3, and CCL4) and is associated with the expression and secretion of active T cells (CCL5) as well as the regulation of CXC chemokine subfamily members. The associations of cytokines and chemokines with TB susceptibility are summarized in Table 3.

## 4. Host Genes with TB in Genome-Wide Association Studies (GWAS)

Several lines of evidence support an important role for host genetic factors in susceptibility to TB disease and MTB infection. However, results across candidate genes and GWAS are largely inconsistent. To date, several GWAS studies have found some host genes. Firstly, the earliest GWAS discovered an association on chromosome 18q11.2 in a combined Ghanaian, Gambian, and Malawian population [79]. Some researchers inputted GWAS SNP data into the Ghanaian cohort and identify a genome-wide significant association for a locus 46 kb downstream of WT1 [80]. Then, this association was replicated in Gambian, Indonesian, and Russian populations and also by an independently conducted GWAS in a mixed population in South Africa [80, 81]. In addition, the South African GWAS also detected loci on chromosomes 14q24.2 and 11q21-q22 [81]. Secondly, a GWAS on Icelandic, Russian, and Croatian populations identified significant association with the HLA region which was consistent with the previous candidate gene studies [82]. Finally, for the latest GWAS of TB which identified a common variant, rs4921437 of the IL12B gene at 5q33.3 was significantly associated with TB in African populations [83].

## 5. Conclusions

Taken together, these studies supported that host genes play an important role for mediating host regulation of MTB infection. The susceptibility of TB is correlated with various genes in multiple loci, and every single gene plays a certain unique role. But most effects in different subjects are inconsistent, which may be influenced by a variety of factors, the diverse source of cases, for example, and the different criteria for inclusion in the case group and the control group. What is more, a small sample size will also have a nonnegligible impact on the results. Therefore, it is necessary to conduct repetitive, multicenter, and large-sample researches in order to draw more scientific conclusions, further to confirm the relationship between TB susceptibility and gene polymorphisms, and to better clarify the immunopathological mechanisms of TB for a theoretical basis for prevention and treatment of TB.

## Figures and Tables

**Table 1:** Summary of association between HLA genes and TB susceptibility.

HLA	Region	HLA antigen allele	Relationship with TB	References
HLA-I gene	Canada	B8	Susceptible	[6]
	India	A2	Susceptible	[7]
		B18	Antagonized	[7]
	Italy	A2, B14	Antagonized	[8]
	Iran	A26, B27	Antagonized	[9]
		B17	Susceptible	[9]
	China	B27, B35	Susceptible	[10]
		A31	Antagonized	[10]

HLA-II gene	Govinda	DRB1∗1302, DQB1∗0301-0304	Susceptible	[11]
	Cambodia	DQ *β*57, DQB1∗0301, -0303, DQB1∗04 (-0401, -0402), DQB1∗0503, DQB1∗0601, -0602, -0603	Susceptible	[12]
	Iran	DRBI∗07, DQA1∗0101	Susceptible	[13]
	Xiangxi, China	DRB1∗09	Susceptible	[14, 15]
		DRB1∗11	Antagonized	
	Xinjiang, China	DRB1∗12	Susceptible	[14, 15]
		DRB1∗11	Antagonized	
	Russia	DRB1∗13	Susceptible	[16]
	India	DQB1∗0503, -0502, DR2, DQB1∗0601, DQ1, DRB1∗1501 (DR2), DRB1∗1501-DRB5∗0101-DQA1∗0103-DQB1∗0601	Susceptible	[4, 17, 18]
		DRW6	Antagonized	[19]
	Poland	DRB1∗16, DQB1∗05, DRB1∗1601-DQB1∗0502, DRB1∗04-DQB1∗03, DRB1∗14-DQB1∗05	Susceptible	[20, 21]
		DRB1∗13	Antagonized	[20]

**Table 2:** Summary of association between the TLR gene and TB susceptibility.

TLR gene	Polymorphic locus	Region	Relationship with TB	References
TLR1	rs4833095 (N248S), rs5743618 (1602S)	Afro-American	Susceptible	[43]

TLR2	rs11938228	Europe, Asia	Susceptible	[44, 45]
		Africa, Spain	Unrelated	
		Asia	Unrelated	[46]
	rs5743708	Asia, Caucasians		[47]

TLR4	rs4986790, rs4986791	India	Susceptible	[48]
		Southeast China, Africa	Unrelated	[46, 49]

TLR8	rs3764879, rs3788935, rs3761624, rs3764880	Indonesia, Russia	Susceptible (male)	[50]

TLR9	rs352143, rs5743836	The U.S. (Caucasian, Black)	Susceptible	[50]

**Table 3:** Association of cytokines, chemokines, and susceptibility to TB.

Cytokine species	Polymorphic locus	Region	Relationship with TB	References
IFN-*γ*	+874A/T	Pakistan, Sicily, South Africa, China, Spain	Susceptible	[57–61]
IFN-*γ*	+874A/T	Turkey, Malawi, Caucasians, West Africa, South India, China	Unrelated	[62–67]
IL-10	-1082	Ghana	Unrelated	[68]
IL-12	Promoter, intron, 3′UTR	Multizone	Inconsistent	[66, 69–72]
IL-6	rs1800796 C allele	China	Susceptible	[73]
IL-18	-137 G/C	China	Susceptible	[74]
IL-22	rs2227472, rs2227473, rs2227478, rs2227483, rs2227485	China	Susceptible	[75]
TNFRSF1B	rs3397	South Africa, Ghana	Antagonized	[76]
CCL2	-2258G/A (rsl024611)	Mexico, Korea	Susceptible	[35, 77]
		Brazil	Unrelated	
CCL5	-28C>G	Asia, Arabs	Antagonized	[78]
